# The endoplasmic reticulum stress induced by tunicamycin affects the viability and autophagy activity of chondrocytes

**DOI:** 10.1002/jcla.23437

**Published:** 2020-06-26

**Authors:** Hao Wu, Zhichao Meng, Yang Jiao, Yali Ren, Xin Yang, Heng Liu, Rui Wang, Yunpeng Cui, Liping Pan, Yongping Cao

**Affiliations:** ^1^ Department of Orthopedics Peking University First Hospital Beijing China; ^2^ Lab of Electron Microscopy Peking University First Hospital Beijing China

**Keywords:** apoptosis, autophagy, chondrocyte, endoplasmic reticulum stress, GRP78

## Abstract

Osteoarthritis (OA) is attributed to a reduction in chondrocytes within joint cartilage, and research has shown that endoplasmic reticulum (ER) stress and autophagy play important roles in the survival of chondrocytes. However, the relationship between ER stress and autophagy in chondrocytes remains unclear. In this study, we investigated the changes in apoptotic and autophagic activity in chondrocytes under ER stress. Following treatment with tunicamycin, the rate of apoptosis among chondrocytes increased. Western blot analysis showed the levels of unfolded protein response (UPR) related proteins increased, followed by elevated expression of light chain 3B‐II (LC3B‐II) and Beclin‐1. An ultrastructural investigation showed that a large number of pre‑autophagosomal structures or autophagosomes formed under tunicamycin treatment. However, the autophagy activity was significantly inhibited in chondrocytes after suppression of GRP78 by siRNA. The apoptosis ratio of chondrocytes pre‐treated with 3‐methyladenine was much higher than that of normal chondrocytes after exposure to tunicamycin. Our study revealed that the tunicamycin‐induced persistent UPR expression led to apoptosis of chondrocytes and activation of autophagy incorporation with GRP78. Blocking autophagy accelerated the apoptosis induced by ER stress, which confirmed the protective function of autophagy in the homeostasis of chondrocytes. These findings advance our understanding of chondrocyte apoptosis and provide potential molecular targets for preventing apoptotic death of chondrocytes.

## Graphical abstract

Fewer autophagosomes had formed in chondrocytes transfected siRNA‐GRP78 than in normal chondrocytes after 24 h of treatment with TM. The results demonstrated that UPR can activate autophagy through the GRP78 pathway to protect chondrocytes from apoptosis. (A) Normal chondrocytes exposed to TM for 24 h and (B) siRNA‐GRP78–transfected chondrocytes exposed to TM for 24 h.

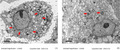

## 1 INTRODUCTION

Osteoarthritis (OA) is the most common joint disease in elderly adults and is characterized by chronic pain and the degradation of articular cartilage. Degradation of extracellular matrix (ECM) and reduced cartilage cellularity are the major histological features of osteoarthritis.^1^, ^2^ The chondrocyte is a cell type unique in cartilage that maintains the dynamic equilibrium between the synthesis and degradation of the ECM, and thus, the normal function and viability of these cells are critical to the prevention of OA.^3^ Therefore, the signaling events that determine the fate and function of chondrocytes, such as the unfolded protein response (UPR) and autophagy, are crucial to the maintenance of healthy cartilage.

The endoplasmic reticulum (ER) plays an important role in the synthesis and assembly of secretary protein. Cellular stress conditions, such as glucose deprivation, oxidative injury, aberrant Ca^2+^ regulation, and the accumulation of unfolded protein, can disrupt proper ER function. To alleviate ER stress and restore normal ER function, the UPR is initiated.^4^ First, with the assistance of protein kinase RNA‐like ER kinase, some translational expression levels are inhibited including the pivotal factor X‐box binding protein 1 (XBP1), which is involved in supporting the cell cycle.^5^, ^6^ Then, the UPR upregulates the expression of chaperone genes to facilitate the degradation of unfolded or misfolded proteins. If the ER stress persists, programmed cell execution is activated via activation of the transcription factor 4 (ATF4)/C/EBP homologous protein C/EBP (CHOP)/growth arrest and DNA damage 34 (GADD34) pathway.^7^ Previous studies have shown that ER stress in the chondrocytes in OA cartilage is related to cartilage degradation.^8^ ER stress can induce the apoptosis of chondrocytes and upregulate the expression of matrix metalloproteinase (MMPs), which degrade the ECM.^9^ Therefore, ER stress is one of the factors that contribute to the development of OA. The natural antibiotic tunicamycin (TM), which inhibits N‐linked glycosylation, can disrupt protein maturation in the ER and has been proposed as an available tool for inducing and studying ER stress.^10^

Autophagy is a cellular response to various stress conditions. During the process, damaged and dysfunctional organelles and molecules are recycled and digested into small molecules that can be used by the cell. Thus, autophagy is considered an essential protective process against cellular damage.^11^ Increased autophagy in response to cellular stress also occurs in chondrocytes, according to evidence showing that autophagy disruption is related to apoptosis of chondrocytes and the development of OA.^12^

Although ER stress and autophagy are functionally independent, these two systems are dynamically connected. They protect cells by relieving stress, but under some extreme conditions, they can both lead to apoptosis.^13^ Furthermore, recent studies have claimed that ER stress can mediate autophagic processes. Using thin section electron microscopy, Bernales et al ^14^ discovered that the UPR in yeasts is accompanied by the formation of autophagosome‐like structures. Margariti et al ^15^ also found that XBP1 mRNA splicing triggers an autophagic response in endothelial cells (ECs) through the transcriptional regulation of Beclin‐1. Moreover, the correlation between ER stress and mitophagy was reported in liver tissue and also shown to have a role in the chondrogenic and osteogenic differentiation of adipose stem cells in equine metabolic syndrome.^16^, ^17^, ^18^ However, these previous studies used neoplastic cells, endothelial cells, or other cells types for their research. To date, only a few studies on ER stress and autophagy in chondrocytes have been published. Yang et al indicated the involvement of the mammalian target of rapamycin (MTOR) complex 1 in coordinating autophagy and ER stress induced by shear stress fluid flow over cultured chondrocytes.^19^ However, the relationship between the TM‐induced ER stress and autophagy in articular chondrocytes remains elusive.

In the present study, we investigated the effect of the UPR induced by TM on the cellular viability of articular chondrocytes. By interfering with the UPR, we also evaluated the relationship between ER stress and autophagy in chondrocytes. Our observations suggest that the UPR induced by TM resulted in the activation of autophagy to protect chondrocytes from apoptosis, but persistent UPR induced the apoptosis of chondrocytes. Additionally, after the suppression of glucose‐regulated protein (GRP) 78, the activation of autophagy induced by UPR was inhibited.

## 2 METHODS

### 2.1 Ethical statement

All protocols and procedures involving animals were performed in accordance with the Regulations for the Administration of Affairs Concerning Experimental Animals (Ministry of Science and Technology, China) and were approved by the Ethics Committee of Peking University 1st Hospital (Beijing, China) (Approval No. J201519). During the experimental period, the rats were reared in the same environment, fed the same diet, and humanely sacrificed.

### 2.2 Chondrocyte isolation and culture

Rat articular cartilage was obtained from the femoral and tibial condyles of normal 4‐ to 6‐week‐old Sprague Dawley male rats. The tissue was cut into 1‐mm^3^ sections and washed in sterilized phosphate‐buffered saline (PBS). These cartilage pieces were digested in trypsin (KeyGen Biotech. Co., Ltd., Nanjing, China) for 30 min and then in collagenase II (Sigma‐Aldrich, St. Louis, MO, USA) for 6 h at 37°C. The digested substrate was filtered and centrifuged (1000 r/min) for cell purification. The chondrocytes were transferred into a 25‐cm^2^ tissue culture flask and cultured in Dulbecco's modified Eagle medium (DMEM) with low glucose (KeyGen Biotech. Co., Ltd., Nanjing, China), 10% fetal bovine serum (FBS) (HyClone, USA), and 100 μg/ml streptomycin and penicillin in 5% CO_2_ incubator a 37°C. Subsequently, the medium was replaced every 2 days. When the cells achieved 70‐80% confluence, the chondrocytes were used for further experiments.

### 2.3 Cell viability assay

The chondrocytes were seeded and cultured in 96‐well plates. After culturing for 24 h, the viability of the chondrocytes was measured every day using a CCK‐8 cell count kit (KeyGen Biotech. Co., Ltd., Nanjing, China). When the chondrocytes were in the logarithmic growth phase (day 4), the cells were divided into two groups: the control (CON) group, which received no treatment, and the TM group, which was treated with 0.5 μg/ml TM (Abcam, Cambridge, MA, USA). This concentration has been widely applied in previous studies to induce ER stress.^20^, ^21^ The differences in the viability between the two groups were compared after treatment for 24, 48, and 72 h. At each time point, 10 μl of CCK‑8 kit solution was added to each well, and the plates were incubated at 37°C for 1 h before cell viability was measured using a microplate reader (#M581789; Westingarea, Shanghai, China).

### 2.4 Apoptosis assay

The chondrocytes were seeded and cultured in 6‐well plates for 4 days before treatment with TM (0.5 μg/ml). Following treatment for 0, 6, 12, and 24 h, the apoptosis ratio of the cells was quantified using an Annexin V‑FITC flow cytometric kit (KeyGen Biotech. Co., Ltd., Nanjing, China) according to the manufacturer's instructions. Briefly, the cells were harvested and washed in PBS three times. Next, the cells were suspended in 500 μl of ice‐cold binding buffer and incubated with 5 μl of Annexin V‑FITC solution and 5 μl of propidium iodide (PI) solution for 30 min. Finally, the apoptosis ratio of the cells was measured using a flow cytometer (BD Biosciences, San Jose, CA, USA). In addition, the apoptotic ratio of chondrocytes pre‐treated with 3‐methyladenine (3‐MA, 2 mM; Santa Cruz Biotechnology, Santa Cruz, CA, USA) for 2 h was quantified after treatment with TM (0.5 μg/ml).

### 2.5 Transmission electron microscopy (TEM) analysis

Normal chondrocytes and those treated with TM (0.5 μg/ml) for the designated times were harvested and washed three times in PBS. The cells were then fixed in ice‐cold 1% glutaraldehyde in 0.1mol/L PBS and preserved for 24 h at 4℃. After fixation, the cells were washed three times in 0.2 M sucrose and then fixed in 1% osmium tetroxide for 1 h at room temperature. Next, all specimens were dehydrated in a graded ethanol series and infiltrated. Then, the cells were embedded in EPON 812. Ultra‐thin sections were cut using an ultramicrotome (Leica, Germany). The sections were stained with 2% uranyl acetate and lead citrate and then viewed and photographed under a transmission electron microscope (JEOL‐1230, Japan).^22^

### 2.6 Western blot analysis

Following treatment with TM (0.5 μg/ml), the cells were harvested at the designated time points, and Western blot analysis was performed as previously described.^23^ In brief, the cells were prepared in lysate buffer containing protease and phosphatase inhibitors. Next, the protein concentration was determined by the Bradford assay. Subsequently, equivalent amounts of protein were resolved on 12% sodium dodecyl sulfate (SDS)‑polyacrylamide gels using the Invitrogen X cell electrophoresis and blotting system (Invitrogen, Carlsbad, CA, USA.). Then, the proteins were electrophoretically transferred to nitrocellulose membranes at 150 V for 1.5 h. The nitrocellulose membranes were blocked with 5% non‑fat milk in wash buffer (Tris‑buffered saline containing 0.1% Tween‑20) for 1 h. The membranes were the incubated with primary antibodies against GRP78 (sc‐13968, 1:1000; Santa Cruz Biotechnology), CHOP (sc‐575, 1:500; Santa Cruz Biotechnology), light chain C3B (LC3B, Sigma L7543, 1:800; Sigma‐Aldrich, St. Louis, MO, USA), ATF4 (cst11815s, 1:1000; Cell Signaling Technology, Boston, MA, USA), GADD34 (10449‐1‐AP, 1:300; Proteintech), and Beclin‐1 (cst3495s, 1:600; Cell Signaling Technology, Boston, MA, USA) at 4℃ for 12 h. After washing 3‐4 times, the membranes were then incubated with secondary antibodies (Santa Cruz Biotechnology) at 1:8000 dilution. Finally, the blots were visualized by chemiluminescence (Millipore, Billerica, MA, USA) using the ProteinSimple Western blot imaging system (Protein Simple, Santa Clara, CA, USA).

### 2.7 siRNA treatment

Three small interfering RNAs (siRNAs) against GRP78 (siRNA‐1: rArGrArUrCrGrGrArGrArUrArArArGrArGrArArGrCrUrGGG, siRNA‐2: rArGrArUrArGrArUrGrUrGrArArUrGrGrUrArUrUrCrUrUCG, and siRNA‐3: rGrUrGrArCrArCrCrArArUrArArArUrGrUrUrUrGrUrUrATT) were designed and synthesized to target GRP78 by Origene Co. Ltd. (USA). Chondrocytes were transfected with the three siRNAs that target GRP78 using Lipofectamine 3000 (Invitrogen) according to the manufacturer's instructions. The chondrocytes were also transfected with a control scrambled siRNA (Origene, USA) targeting a sequence that shared no homology with the rat genome (negative control, NC). Western blot analysis was used to evaluate the suppression rates of the siRNAs after transfection for 24 h. Then, following 24 h treatment with TM, the expression levels of two proteins related to autophagy (LC3B and Beclin‐1) were analyzed by western blotting, and autophagosome formation was observed via TEM as described above.

### 2.8 Statistical analyses

The data are reported as mean ± standard error of the mean (SEM) values. One‐way analysis of variance (ANOVA) was used to analyze values among multiple groups, and Student's t test was used to compare values between two groups. All data were analyzed using SPSS 19.0 software (SPSS Inc, Chicago, IL, USA), and the figures were generated using GraphPad Prism (GraphPad Software Inc, La Jolla, CA, USA). A level of *P* < .05 was accepted as statistically significant.

## 3 RESULTS

### 3.1 ER stress induced by TM decreased the viability of chondrocytes and increased the rate of apoptosis

**FIGURE 1:**
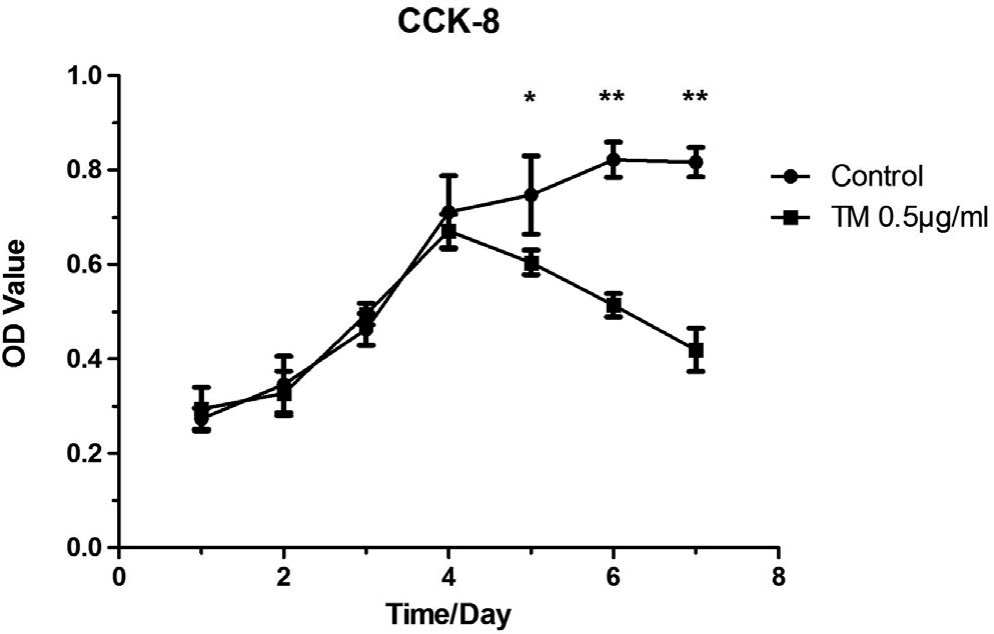
Proliferation of chondrocytes in CON and TM groups. TM was added to chondrocytes of the TM group on day 4 in culture. After different durations of TM treatment, the viability index of the chondrocytes in each group was assessed using a CCK‐8 assay. *P < .05; **P < .01

**FIGURE 2:**
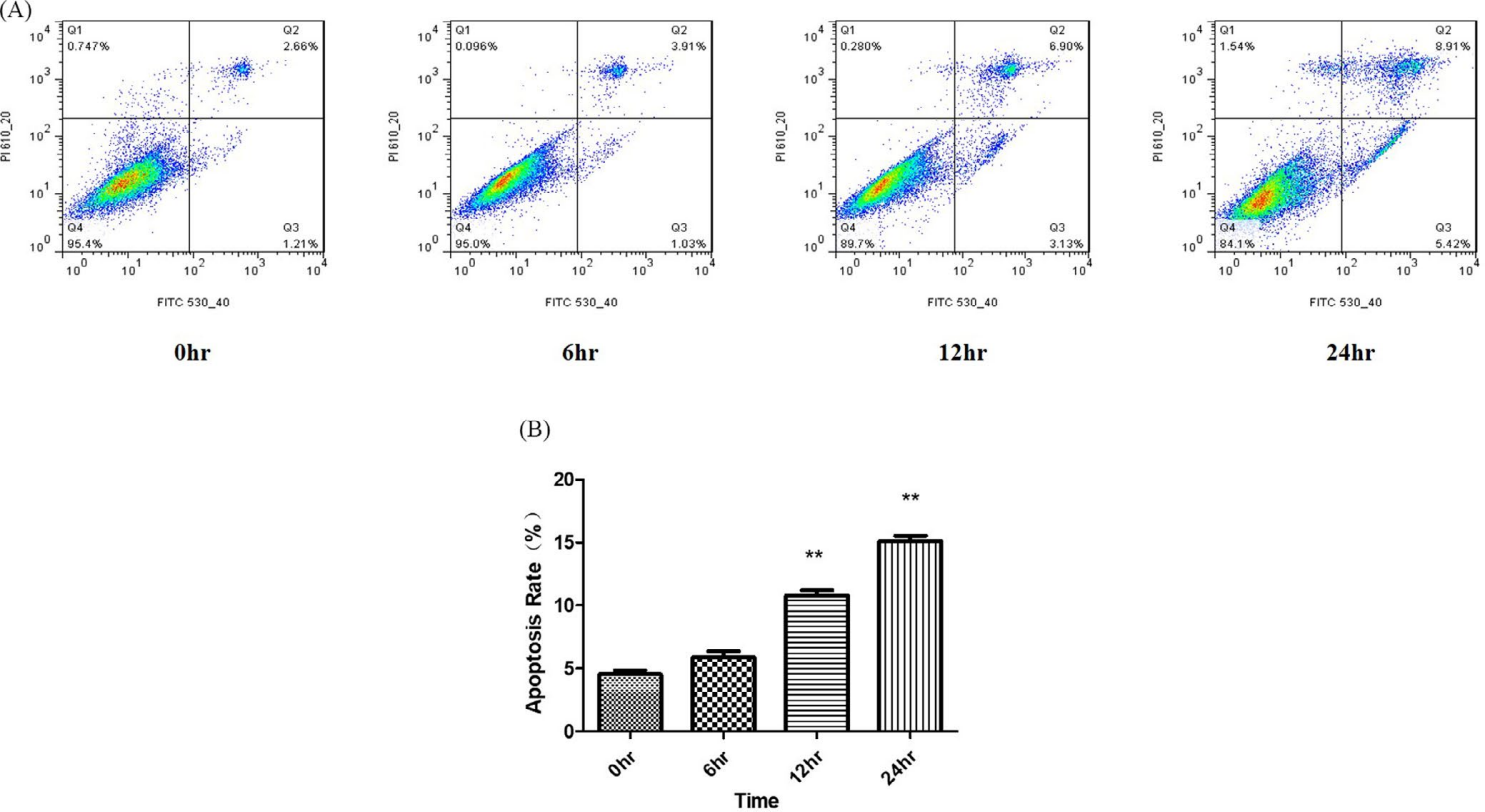
Apoptosis of chondrocytes with increasing durations of TM treatment. The apoptosis rate among chondrocytes increased in a time‐dependent manner in response to TM treatment

The viability of chondrocytes increased obviously during the first 4 days in culture. However, after exposure to TM (0.5 μg/ml) for 72 h, the viability of the chondrocytes was significantly less than that in the CON group (*P < .05 or **P < .01; Figure 1). To further determine the cytotoxicity of TM in chondrocytes, we accessed the rate of apoptosis using Annexin V‐FITC staining and flow cytometry. As shown in Figure 2, following 12‐24 h of TM treatment, the apoptosis rate of chondrocytes increased rapidly (Figure 2). These results indicated that the ER stress induced by TM decreased chondrocyte viability and increased the rate of apoptosis in a time‐ and dose‐dependent manner.

### 3.2 The ER expanded during induction of the UPR, which was accompanied by autophagosome formation

**FIGURE 3:**
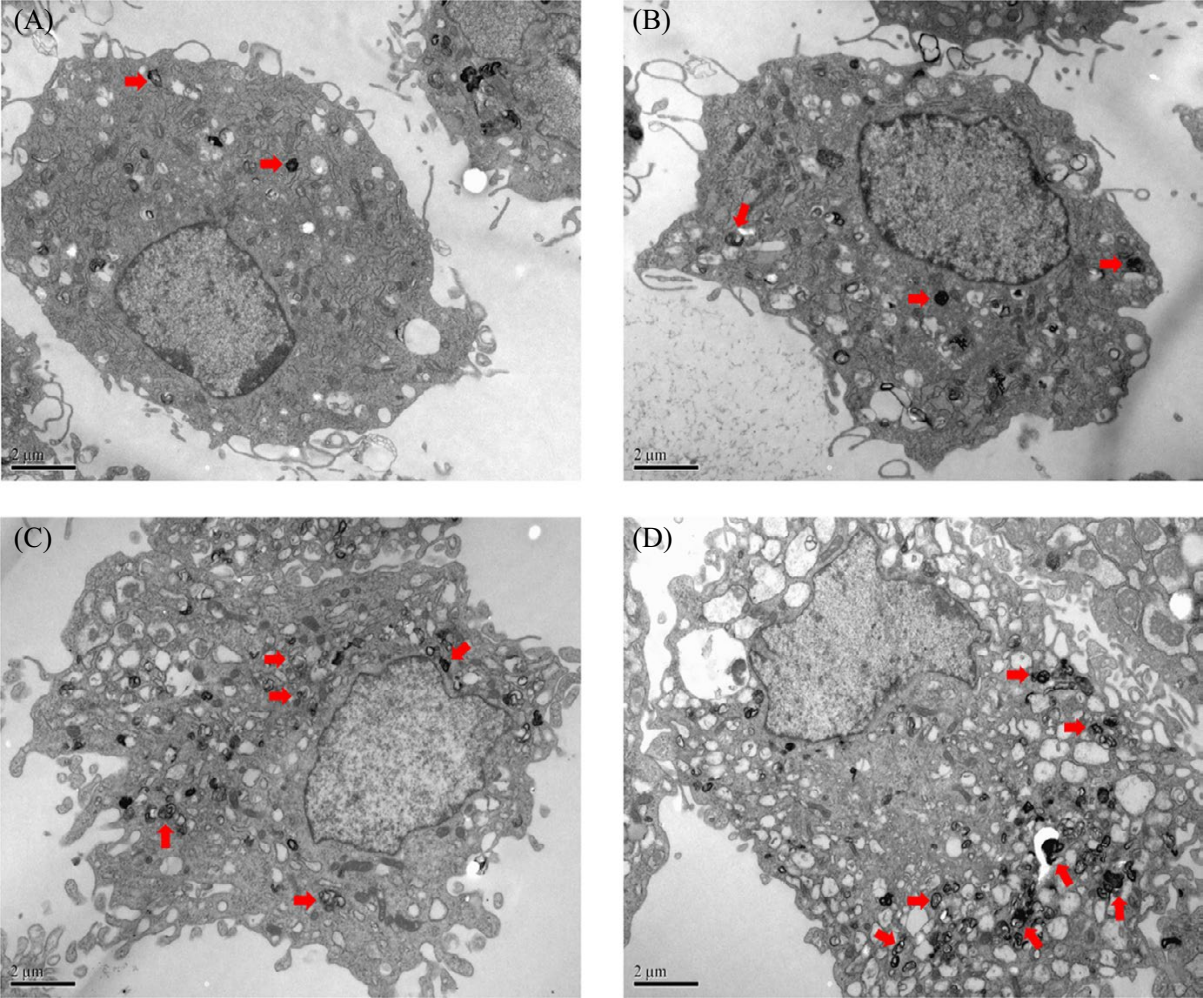
Formation of autophagosomes in chondrocytes treated with TM. Formation of autophagosomes (marked by black arrows) in chondrocytes during TM treatment was observed by TEM (×10 000). Shown are representative images taken at: (A) 0 h (control group); (B) 6 h; (C) 12 h; and (D) 24 h

To determine the ultrastructural changes in chondrocytes treated with TM, we observed the cellular morphology by TEM. As shown in Figure 3, massive expansion of the ER occurred following treatment with TM (0.5 μg/ml), especially after 12‐24 h of treatment. Unexpectedly, during the induction of the UPR, large amounts of autophagosomes with a high electron density accumulated in the cells. As shown in Figure 3, the number of autophagosomes increased after 12 h, and some undegraded substrates were contained within the autophagosomes, which may indicate the formation of mature autophagosomes and an increase in autophagy activity.^24^

### 3.3 TM induced the expression of proteins related to the UPR (GRP78, ATF4, CHOP, and GADD34) and autophagy (LC3B and Beclin‐1)

**FIGURE 4:**
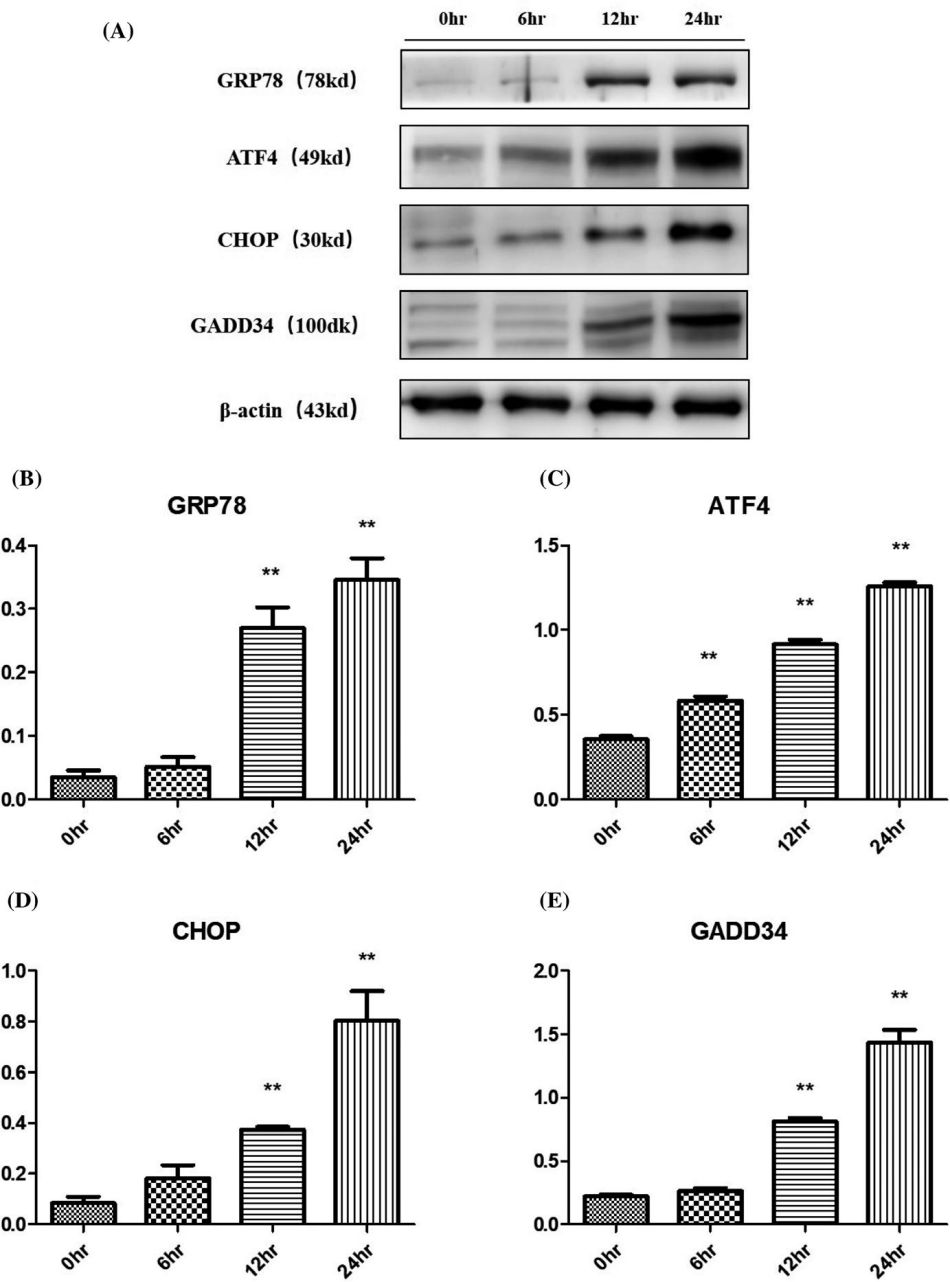
Expression of proteins related to the UPR in chondrocytes during 24 h of TM treatment. Over 24 h of treatment with TM, the expression levels of proteins related to the UPR in chondrocytes increased significantly in a time‐dependent manner. *P < .05 vs 0 h; **P < .01 vs 0 h

**FIGURE 5:**
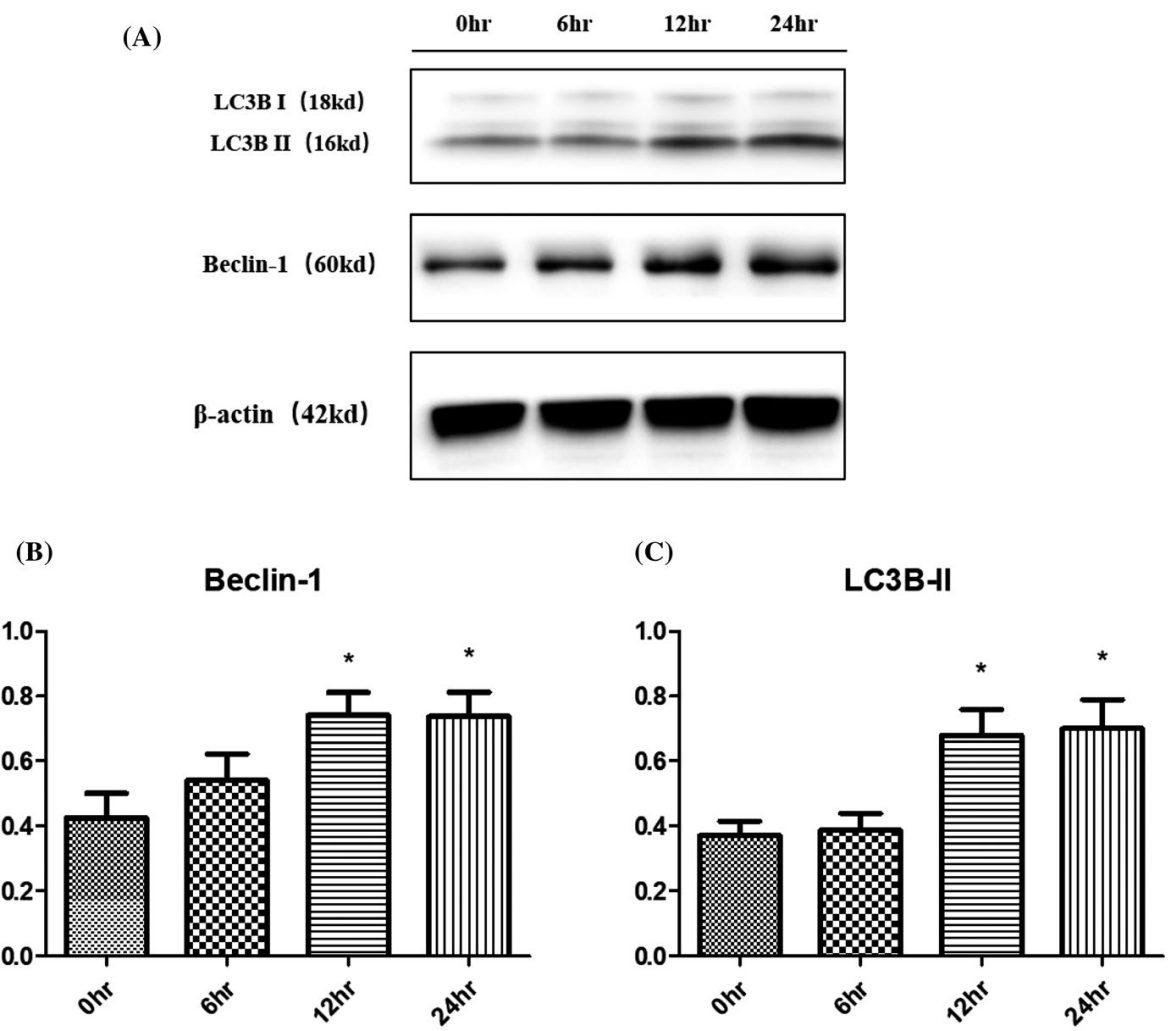
Expression of proteins related to autophagy in chondrocytes during 24 h of TM treatment. Over 24 h of treatment with TM, the expression levels of proteins related to autophagy in chondrocytes changed significantly. *P < .05 vs 0 h; **P < .01 vs 0 h

Western blot analysis showed that the protein levels of UPR‐related regulators, including GRP78, ATF4, CHOP, and GADD34, were low in the normal chondrocytes (Figure 4). Intriguingly, the expression levels of these four proteins in chondrocytes were all significantly increased after TM treatment for 24 h (*P < .05 or **P < .01). At the same time, the expression level of Beclin‐1, which is related to the nucleation of autophagic vesicles, was significantly increased after TM treatment for 12‐24 h (*P < .05). Meanwhile, a rapid increase in LC3B‐II expression was seen after 12‐24 h of TM treatment (Figure 5). The Western blot results indicated that TM induced the UPR as well as an increase in the autophagy activity in chondrocytes in vitro.

### 3.4 Overactivation of the UPR induced by TM attenuated the autophagy activity in chondrocytes after deprivation of GRP78

**FIGURE 6:**
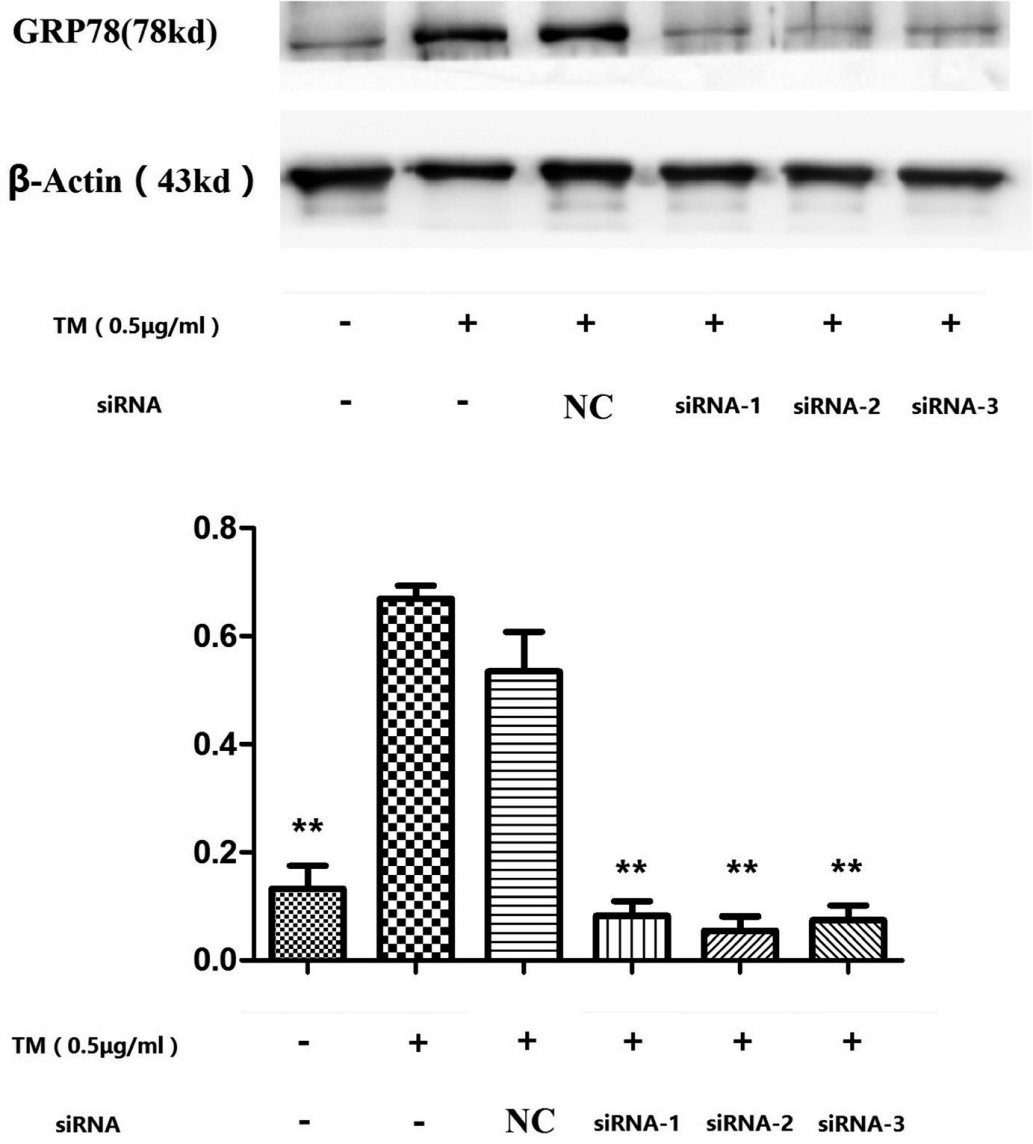
Expression of GRP78 in normal chondrocytes and cells transfected with siRNA after 24 h of TM treatment. After 24 h of treatment with TM, the expression of GRP78 in chondrocytes transfected with each of the three siRNAs for GRP78 was decreased significantly compared to that in normal chondrocytes. Among the three siRNAs, the highest efficiency of GRP78 silencing was achieved with siRNA‐2. **P < .01 vs TM+/siRNA‐ group

**FIGURE 7:**
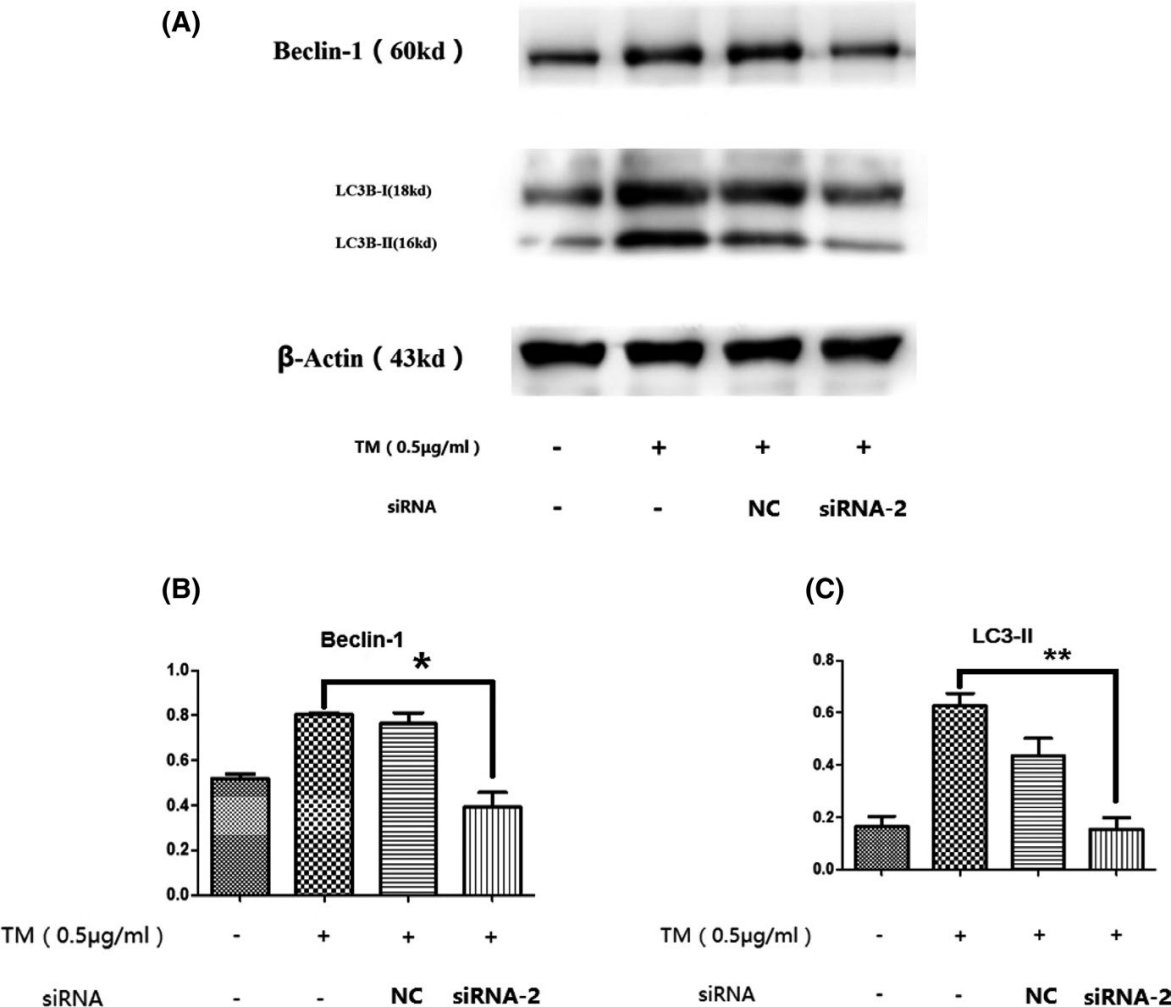
Expression of autophagy‐related proteins was decreased in siRNA‐GRP78 transfected chondrocytes compared with normal chondrocytes after 24 h of TM treatment. After 24 h of treatment with TM, the protein expression levels of LC3B‐Ⅱ and Beclin‐1 in chondrocytes transfected with siRNA‐2 were much lower than those in normal chondrocytes. *P < .05 vs TM+/siRNA‐ group; **P < .01 vs TM+/siRNA‐ group

**FIGURE 8:**
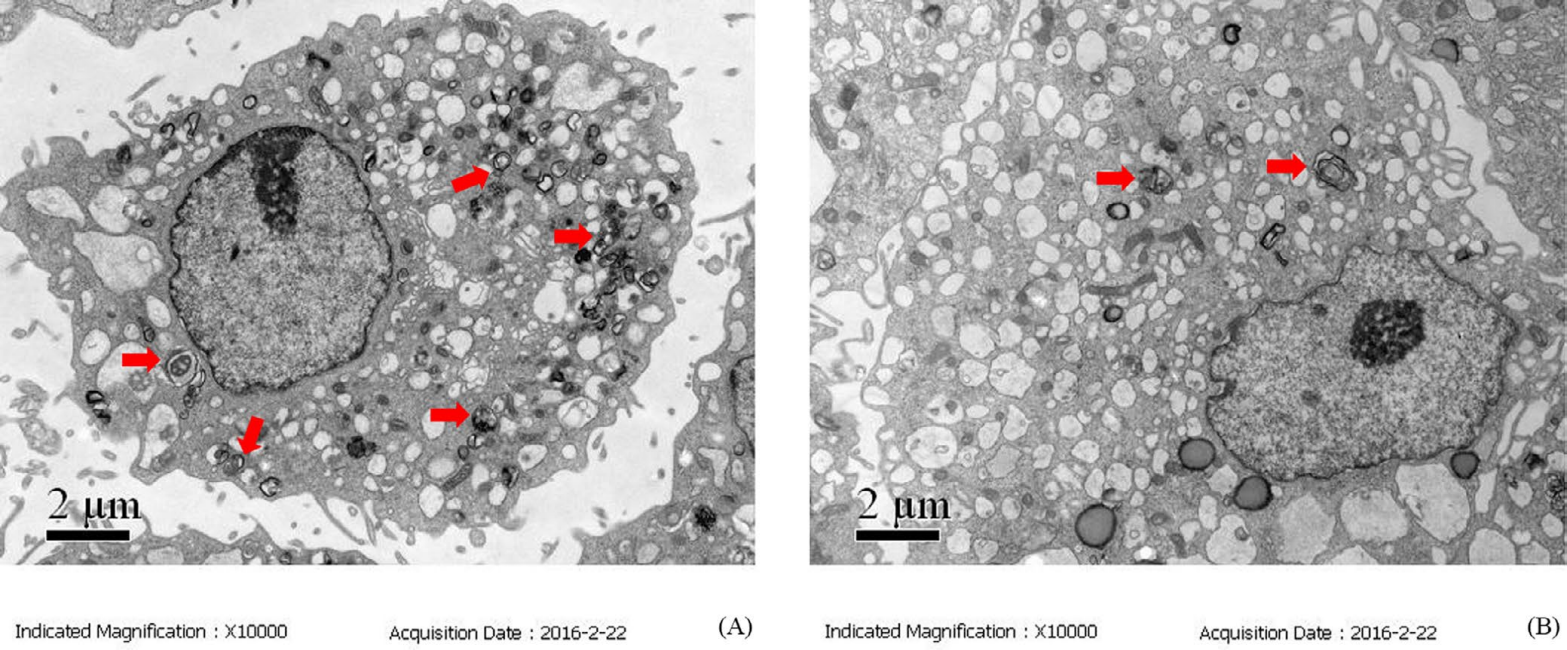
Autophagosome formation in transfected chondrocytes. Fewer autophagosomes had formed in chondrocytes transfected siRNA‐GRP78 than in normal chondrocytes after 24 h of treatment with TM. (A) Normal chondrocytes exposed to TM for 24 h; (B) siRNA‐GRP78–transfected chondrocytes exposed to TM for 24 h

In this study, we examined the effect of the UPR on autophagy induced by TM after knockdown of GRP78, which is an important regulator of the UPR. Among three siRNAs targeting GRP78, siRNA‐2 showed the highest inhibition efficiency and was chosen for use in further experiments (Figure 6). After 24 h of treatment with TM, the protein levels of LC3B‐Ⅱ and Beclin‐1 in chondrocytes transfected with siRNA‐2 were much lower than those in the cells transfected with control scrambled siRNA (*P < .05 or **P < .01, Figure 7). Consistently, we also observed less autophagosome formation in transfected chondrocytes than in normal cells after 24 h of treatment (Figure 8). These results indicate that the upregulation of GRP78 played an important role in the activation of autophagy in chondrocytes during the persistent UPR induced by TM.

### 3.5 Autophagy is induced during ER stress to protect against cell death

**FIGURE 9:**
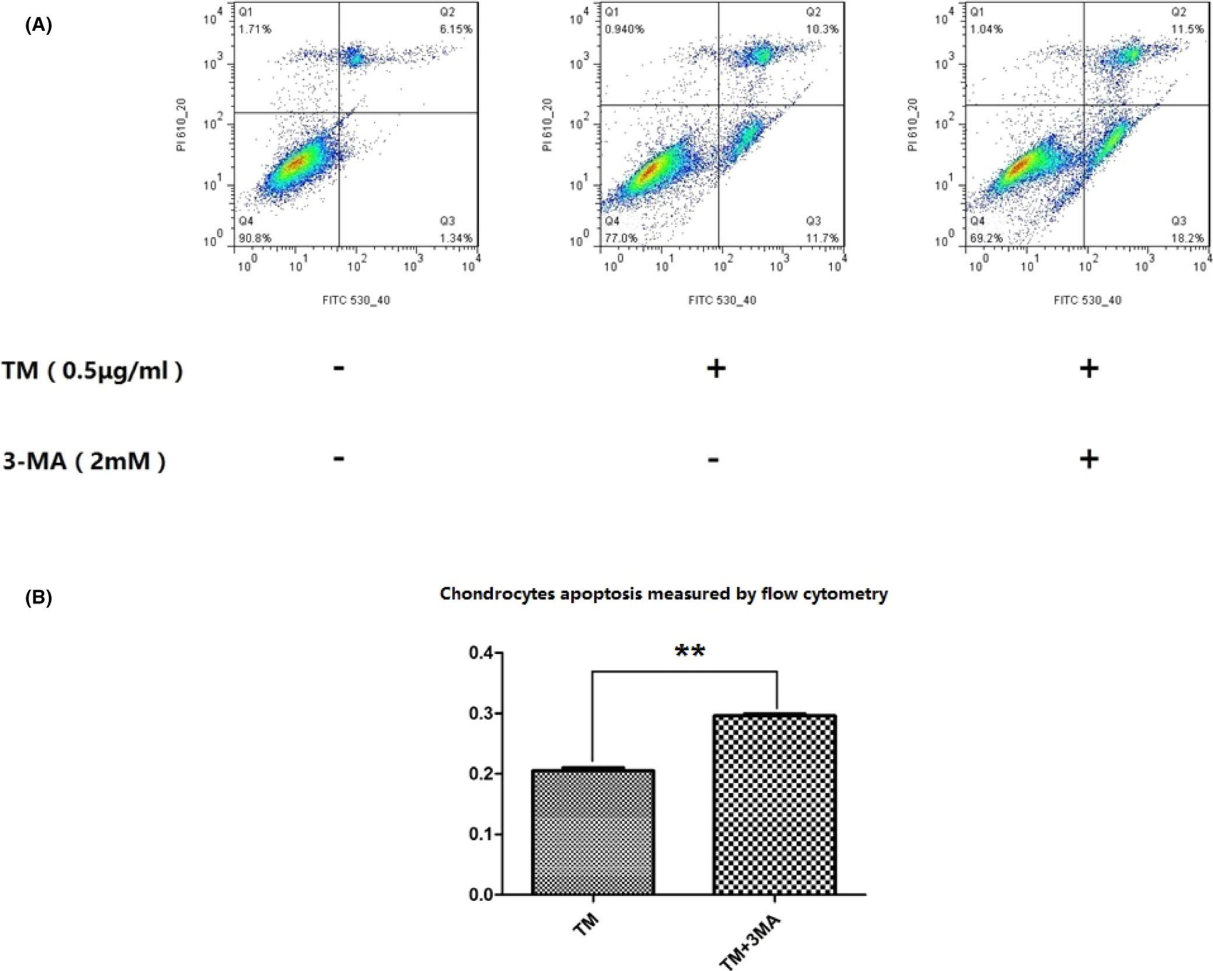
Pre‐treatment with 3‐MA led to increased apoptosis among TM‐treated chondrocytes. After 24 h of treatment with TM, the apoptosis rate among chondrocytes pre‐treated with 3‐MA was much higher than that among normal chondrocytes. **P < .01

After 24 h of treatment with TM, the apoptosis rate among chondrocytes that had been pre‐treated with 3‐MA was much higher than that among normal chondrocytes (**P < .01; Figure 9). This result confirmed the protective effect of autophagy in chondrocytes exposed to TM.

## 4 DISCUSSION

The ER is a vast membranous network that serves as the major assembly and folding site for almost all secreted and integral membrane proteins. Furthermore, the ER is involved in maintaining intracellular Ca^2+^ equilibration as well as cellular signal reception, transmission, and responses.^25^ Many studies have shown that ER stress induces cell function changes and apoptosis that contribute to a variety of degenerative diseases, such as diabetes,^26^ congestive cardiomyopathy,^27^ Parkinson's disease,^28^ and OA. The occurrence and development of OA are related to ER stress specifically in chondrocytes. Previous studies have observed that excessive ER stress induces apoptosis of chondrocytes in vitro.^29^ Liu et al^30^ also proved that ER stress is highly involved in TM‐induced apoptosis in chondrocytes, according to the ability of tauroursodeoxycholic acid to prevent this effect. Another study demonstrated that activating transcription factor 6 (ATF6) and inositol‐requiring enzyme‐1 (IRE1α) can regulate the expression of endogenous XBP1S, which inhibits chondrocyte apoptosis in OA by altering the expression of caspase 3, caspase 9, caspase 12, p‐JNK1, and CHOP.^31^

In our research, ER stress induced by TM treatment reduced the viability and increased apoptosis of chondrocytes in vitro, consistent with the findings of previous studies. In addition, we observed that the apoptosis of chondrocytes induced by TM was promoted by 3‐MA, which is an autophagy inhibitor. These results indicate that autophagy, a common process in eukaryotic cells,^32^ plays an important role in protecting chondrocytes from apoptosis during ER stress. In another experiment, autophagic vesicles and the expression levels of autophagy‐related 5 (ATG‐5) and LC3 decreased with increasing age, followed by a reduction in the density of chondrocytes in cartilage and an increase in the apoptosis rate among chondrocytes.^33^ Rapamycin, an inducer of autophagy, was shown to reduce the severity of experimental OA when administered via intraperitoneal injection.^34^ Therefore, toward the discovery of a new approach to the early prevention of OA, it is meaningful to study the relationship between ER stress and autophagy in chondrocytes.

GRP78, a molecular chaperone, binds transmembrane protein, such as IRE1α, protein kinase R‐like ER kinase (PERK), and ATF6, to prevent activation of the UPR under physiological conditions. Under pathological conditions, such as aberrant Ca^2+^ regulation, hypoxia, and mutant protein accumulation, GRP78 binds to unfolded proteins and initiates the UPR to relieve ER stress.^4^ In our experiment, the expression of GRP78 in chondrocytes was increased significantly following 12 h of treatment with TM. Increased expression of ATF4, a downstream protein of PERK, was also observed in our study. In addition to upregulation of GRP78 and ATF4, the expression levels of CHOP and GADD34, which are involved in apoptosis, also showed rapid increases. At the same time, the protein levels of LC3B‐II and Beclin‐1, markers of autophagy, were increased after TM treatment for 12‐24 h. Because the comparison of LC3‐I and LC3‐II may not totally indicate autophagic flux,^35^ we also observed the formation of autophagosomes in chondrocytes via TEM. We found that the number and volume of autophagosomes increased with ER expansion during TM treatment in a time‐dependent manner. To further explore the connection between ER stress and autophagy, we used an siRNA targeting GRP78 to overactivate the UPR. Following transfection with siRNA‐GRP78, the number of autophagosomes and the expression levels of LC3B‐Ⅱ and Beclin‐1 in chondrocytes were reduced significantly during ER stress.

These results indicate that the UPR induced by TM led to the activation of autophagy in normal chondrocytes. Recent studies have demonstrated that ER stress can induce autophagy in other mammalian cells. The correlation between autophagy activation and ER expansion caused by ER stress was first described in 2006.^36^ It was reported that autophagosome formation, Beclin‐1 expression, and LC3‐II conversion increase in rheumatoid arthritis synovial fibroblasts following thapsigargin treatment.^37^ Bhavya et al^38^ found that TM treatment leads to upregulation of the ER chaperone Grp78 and activates the autophagy pathway in renal tubular epithelial cells, and these effects were beneficial in ischemia‐reperfusion injury both in vitro and in vivo.

In our experiment, with the suppression of GRP78, the activation of autophagy induced by the UPR was inhibited. Therefore, our results indicate that overexpression of GRP78 under ER stress may activate autophagy to protect chondrocytes. A previous study conducted by Zhang et al also concluded that upregulation of GRP78 may activate autophagy through the AMP‐activated protein kinase (AMPK)‐mammalian target of rapamycin (mTOR) pathway in neural cells under extreme conditions.^39^ However, the intracellular pathways connecting ER stress and autophagy are very complicated and remain unclear. It has been reported that the eukaryotic initiation factor 2 alpha (eIF2α)‐ATF4 pathway has a novel regulatory role in autophagy gene transcription in response to stress in fibroblasts.^40^ Kouroku et al^41^ revealed that PERK/eIF2α phosphorylation is involved in polyglutamine‐induced LC3 conversion and that autophagy activation protects cells from ER stress‐mediated cell death by degrading polyglutamine aggregates. The IRE1α‐JNK1 pathway is also a major regulator of autophagy activation. Prior research revealed that JNK1 phosphorylates BCL2 to disrupt the interaction of Beclin‐1 and BCL2, which induces autophagy activation in tumor cells.^42^ In addition, CHOP overexpression during ER stress causes down‐regulation of BCL2 in tumor cells.^43^

In conclusion, the present study demonstrated that treatment of chondrocytes with TM to induce ER stress resulted in activation of the UPR, and persistent UPR induced apoptosis among the chondrocytes. Our experiments also confirmed that the UPR can activate autophagy through the GRP78 pathway to protect chondrocytes from apoptosis.

## CONFLICTS OF INTEREST

All authors have no conflicts of interest.

## AUTHORS' CONTRIBUTION

Study design: Hao Wu, Zhichao Meng, and Yongping Cao. Experiment completion: Hao Wu, Yang Jiao, Yali Ren, and Liping Pan. Analysis of data: Hao Wu, Zhichao Meng, Xin Yang, and Heng Liu. Critical revision of the paper: Yongping Cao, Xin Yang, Rui Wang, and Yunpeng Cui. All authors read and approved the final version of the manuscript.
